# Amino Acid and Lipid Metabolism in Cancer: Mechanisms and Therapeutic Opportunities

**DOI:** 10.1002/mco2.70886

**Published:** 2026-07-19

**Authors:** Zixu Wang, Xin Fu, Jingchao Bai, Yanguo Liu, Kai Huang

**Affiliations:** ^1^ Department of Medical Oncology Qilu Hospital of Shandong University Jinan China; ^2^ Department of Gynecologic Oncology Tianjin Medical University Cancer Institute and Hospital Tianjin China; ^3^ Department of Cancer Biology Perelman School of Medicine University of Pennsylvania Philadelphia Pennsylvania USA

**Keywords:** amino acid metabolism, cancer immunotherapy, immune evasion, lipid metabolism, metabolic reprogramming

## Abstract

Metabolic reprogramming is a defining feature of cancer and a major contributor to immune escape. Beyond the well‐defined glycolysis, dysregulated amino acid and lipid metabolism also regulate tumor growth, stress adaptation, and therapeutic resistance. Amino acids such as glutamine, arginine, tryptophan, methionine, serine, and cysteine shape biosynthesis, redox balance, one‐carbon metabolism, epigenetic control, and nutrient competition in the tumor microenvironment. Lipid uptake, de novo lipogenesis, fatty acid oxidation, cholesterol remodeling, COX‐PGE2 signaling, sphingolipid metabolism, and ferroptosis further influence antigen presentation, immune cell fitness, and checkpoint regulation. However, most studies still consider these metabolic axes separately, leaving the coordinated amino acid–lipid crosstalk across tumor and immune compartments insufficiently defined. This review synthesizes recent advances in amino acid metabolism, including the glutamine axis, arginine‐polyamine biology, the tryptophan–kynurenine–AHR pathway, and methionine‐dependent methylation programs. It then discusses lipid metabolic programs that regulate dendritic cell cross‐presentation, suppressive myeloid polarization, CD8^+^ T cell exhaustion, PD‐L1 palmitoylation, MHC‐I stability, and lipid‐peroxidation‐linked ferroptosis. We further integrate nutrient competition, immunometabolic checkpoints, and dual metabolic targeting strategies with immune checkpoint blockade. This review provides a unified framework for identifying metabolic vulnerabilities, designing rational combination therapies and refining precision cancer immunotherapy.

## Graphical abstract

*Amino Acid and lipid metabolism in cancer*: (A) Regulation of Amino acid transportation and metabolism in cancer. (B) The core organization of lipid metabolic reprogramming in cancer and their functional consequences.

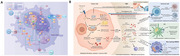

## 1 Introduction

Cancer cells undergo profound metabolic reprogramming to sustain their rapid proliferation, survival under stress, and immune evasion [1]. The altered utilization of nutrients, particularly amino acids and lipids, are among the most important features. They act as fundamental building blocks of macromolecules as well as signaling mediators that influence tumor progression and therapeutic response [2]. Early studies on the “Warburg effect” revealed that tumor cells rely heavily on aerobic glycolysis [3]; however, accumulating evidence over the past two decades has highlighted that alterations of amino acid and lipid metabolism is equally essential in shaping the tumor microenvironment (TME) [4, 5]. These metabolic networks provide cancer cells with biosynthetic precursors, maintain redox homeostasis, and generate metabolites that regulate gene expression and immune function, thereby positioning them as central drivers of tumorigenesis. In this context, amino acid and lipid metabolism can be defined as interconnected biochemical networks governing nutrient utilization, biosynthesis, and signaling, with key milestones including the identification of glutamine addiction in cancer and the recognition of lipid‐driven immune suppression in the TME.

Amino acid metabolism plays a fundamental role in cellular function, supporting processes such as protein synthesis, energy production, and cell signaling [6, 7]. In cancer, metabolic reprogramming allows tumor cells to acquire and utilize amino acids in ways that promote survival, proliferation, and immune evasion [8, 9]. As a result, alterations in amino acid metabolism are increasingly recognized as key hallmarks of cancer and are being actively explored as therapeutic targets [10]. Within the TME, amino acid metabolism influences both cancer cells and immune cells, creating a dynamic interplay that can either promote or suppress antitumor immunity. Tumor cells often hijack metabolic pathways to secure a competitive advantage, and deprive immune cells of essential nutrients while simultaneously producing immunosuppressive metabolites [11, 12]. This metabolic competition contributes to immune cell dysfunction, facilitating tumor progression and resistance to therapy. Additionally, distinct immune cell populations, such as T cells, macrophages, and myeloid‐derived suppressor cells (MDSCs), rely on specific amino acids for activation and function, further underscoring the importance of metabolic regulation in shaping immune responses [13, 14, 15].

Aside from amino acids, lipid metabolism has emerged as another critical regulator of tumor progression. Cancer cells exhibit enhanced lipid uptake, de novo synthesis, and storage to support membrane biogenesis, energy production, and survival under nutrient‐limited conditions [16]. Fatty acid oxidation (FAO) provides an efficient ATP source and contributes to therapy resistance, while altered cholesterol and sphingolipid metabolism promote oncogenic signaling and metastasis [17]. Dysregulated lipid metabolism also induces immunomodulatory effects: lipid accumulation in T cells promotes functional exhaustion, lipid‐driven polarization of macrophages fosters immunosuppression, and lipid overload in dendritic cells (DCs) impairs antigen presentation [18, 19, 20]. These findings underscore the dual role of lipid metabolism in fueling tumor progression while simultaneously sabotaging immune surveillance.

Given the importance of both amino acid and lipid pathways, it is necessary to have an integrated point of view on their functions. This review aims to provide a structured and integrated overview of amino acid and lipid metabolism in cancer, clarify their coordinated roles in shaping tumor–immune interactions, and outline emerging therapeutic strategies based on dual metabolic targeting.

## 2 Amino Acid Metabolism in Cancer

Under normal physiological conditions, amino acid metabolism is tightly regulated to maintain cellular homeostasis and support essential biological processes. Amino acids serve both as building blocks for protein synthesis and key substrates for energy production, biosynthetic pathways, and signaling networks [21]. Through interconnected pathways such as transamination, deamination, and the tricarboxylic acid (TCA) cycle, amino acids contribute to the generation of metabolic intermediates required for nucleotide synthesis, lipid metabolism, redox balance, and epigenetic regulation [22, 23]. In addition, amino acid availability is dynamically controlled by dietary intake, intracellular synthesis, and transport systems that regulate uptake and exchange between tissues [24, 25]. This coordinated metabolic network allows cells to adapt to changing nutrient conditions while maintaining balanced growth, metabolism, and immune function (Table 1).

**TABLE 1:** Transport and metabolism of major amino acids in TME.

Amino acids	Transporter	Aliases	Enzyme	Main substrate	Main products	Key refs
Gln	SLC1A2	EAAT2	GS	Glu	Gln	[24– 28]
	SLC1A3	EAAT1	GLS	Gln	Glu	
	SLC1A5	ASCT2	ASNS	Gln/Asp	Glu/Asn	
	SLC3A2	CD98				
	SLC7A5	LAT1				
	SLC7A11	XCT				
	SLC38A1	SNAT1				
	SLC38A2	SNAT2				
Arg	SLC7A1	CAT1	ARG	Arg	Orn/Urea	[24, 29, 30, 31]
	SLC7A2	CAT2	NOS	Arg、NADPH	NO/Citrulline	
			ASS	Citrulline/Asp	Argininosuccinic acid	
			ASL	Argininosuccinic acid	Arg/allomaleic acid	
Trp	SLC7A5	LAT1	IDO1/2	Trp	Kyn	[32, 33, 34, 35]
			TPH	Trp	5‐HTP	
			TDO	Trp	Kyn	
Ser	SLC6A14		PHGDH	3‐PG	3‐PHP	[36, 37, 38]
	SLC12A4		PSAT1	3‐PHP/Glu	α‐KG/PSer	
	SLC25A15		PSPH	PSer	Ser	
Met	SLC7A5	LAT1	MAT	Met	SAM	[36, 39, 40, 41]
	SLC38A2	SNAT2				

Abbreviations: α‐KG, α‐ketoglutarate; 3‐PG, 3‐phosphoglycerate; 3‐PHP, 3‐phosphohydroxypyruvate; 5‐HTP, 5‐hydroxytryptophan; AAs, amino acids; Arg, arginine; ASCT2, alanine‐serine‐cysteine transporter 2; ASL, argininosuccinate lyase; Asp, aspartate; ASS, argininosuccinate synthase; CAT1/2, cationic amino acid transporter 1/2; CD98 (SLC3A2), 4F2 heavy chain; EAAT1/2, excitatory amino acid transporter 1/2; Gln, glutamine; GLS, glutaminase; Glu, glutamate; GS, glutamine synthetase; Kyn, kynurenine; LAT1, l‐type amino acid transporter 1; MAT, methionine adenosyltransferase; Met, methionine; NADPH, nicotinamide adenine dinucleotide phosphate (reduced form); NO, nitric oxide; NOS, nitric oxide synthase; Orn, ornithine; PHGDH, phosphoglycerate dehydrogenase; PSAT1, phosphoserine aminotransferase 1; PSer, phosphoserine; PSPH, phosphoserine phosphatase; SAM, S‐adenosylmethionine; Ser, serine; SNAT1/2, sodium‐coupled neutral amino acid transporter 1/2; Trp, tryptophan; XCT (SLC7A11), cystine/glutamate antiporter.

### 2.1 Reprogramming of Key Amino Acid Pathways in Tumors

**FIGURE 1:**
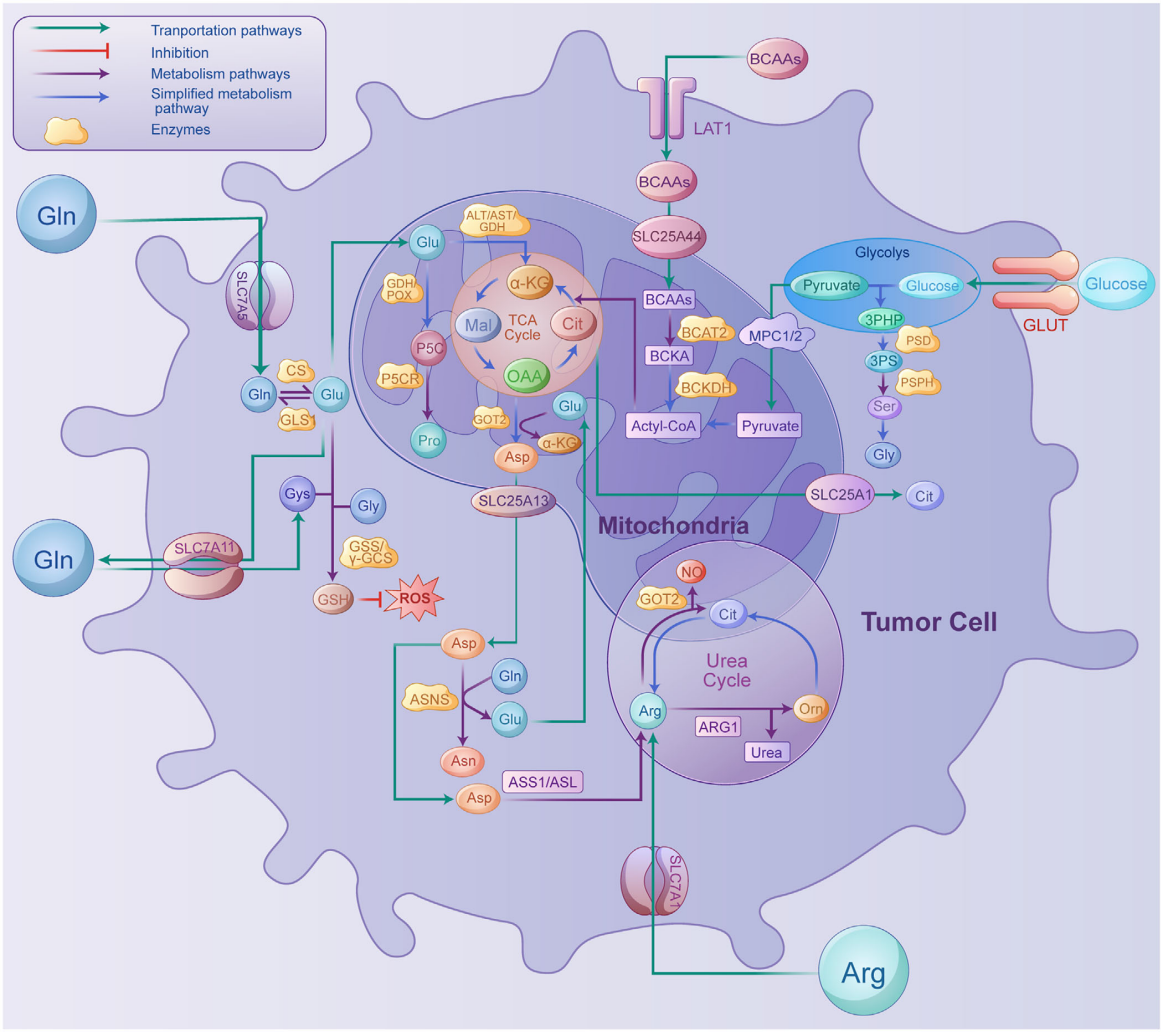
Major amino acid metabolic pathways and metabolic flexibility in tumor cells. The uptake, transport, and interconversion of major amino acids in tumor cells, including glutamine, arginine, serine, and branched‐chain amino acids. These pathways connect with the TCA cycle, urea cycle, glycolytic intermediates, and glutathione synthesis to support biosynthesis, redox balance, and adaptation to nutrient stress in the TME. α‐KG, α‐ketoglutarate; ALT, alanine transaminase; Arg, arginine; ASL, argininosuccinate lyase; ASNS, asparagine synthetase; ASS1, argininosuccinate synthase 1; AST/GOT2, aspartate transaminase/glutamic‐oxaloacetic transaminase 2; BCAT2, branched‐chain amino acid transaminase 2; BCKDH, branched‐chain ketoacid dehydrogenase; GDH, glutamate dehydrogenase; Gln, glutamine; GLS1, glutaminase 1; Leu, leucine; Met, methionine; OAA, oxaloacetate; ROS, reactive oxygen species; TCA, tricarboxylic acid; Trp, tryptophan.

In cancer, amino acid metabolism undergoes extensive reprogramming to support rapid proliferation and adaptation to the harsh TME [42, 43, 44]. This section highlights key amino acid metabolic pathways and their implications in cancer biology (Figure 1).

### 2.2 Glutamine

Glutamine is a central metabolic hub in cancer cells, fueling multiple biosynthetic and energy‐generating pathways. It is converted to glutamate by glutaminase (GLS), which is often upregulated in tumors under oncogenic control, such as MYC amplification [26, 45]. Glutamate is further metabolized into α‐ketoglutarate (α‐KG), an essential intermediate in the TCA cycle that supports ATP production, lipid synthesis, and nucleotide biosynthesis [46]. Additionally, glutamine‐derived glutathione (GSH) plays a key role in maintaining redox balance, protecting cancer cells from oxidative stress [46, 47, 48, 49]. This metabolic dependence has been reported in multiple tumor types, including triple‐negative breast cancer and HER2‐positive gastric cancer.

Tumor cells exhibit a high demand for glutamine, facilitated by increased expression of transporters such as SLC1A5 (ASCT2) and SLC7A5 (LAT1). This competition for glutamine impacts immune cells, as its depletion in the TME weakens T cell proliferation and effector functions [27, 50, 51, 52]. Targeting glutamine metabolism with GLS inhibitors (e.g., CB‐839) or glutamine transport inhibitors (e.g., V9302) has emerged as a promising strategy to selectively starve tumor cells while restoring immune function [28, 53, 54].

### 2.3 Arginine

Arginine serves as a precursor for nitric oxide (NO), polyamines, and creatine, playing crucial roles in tumor progression and immune regulation [55]. Its metabolism is tightly controlled by enzymes such as argininosuccinate synthase 1 (ASS1) and arginase (ARG) [42]. Many tumors downregulate ASS1 expression, therefore becoming dependent on extracellular arginine uptake via cationic amino acid transporters (CATs) such as SLC7A1 in liver cancer and colorectal cancer [29, 56, 57, 58]. Conversely, high expression of ARG1 in MDSCs depletes arginine in the TME, impairing T cell proliferation and promoting an immunosuppressive environment [59].

Therapeutic strategies targeting arginine metabolism include ARG inhibitors (e.g., CB‐1158) and arginine‐depleting enzymes (e.g., pegylated arginine deiminase), aiming to restore immune competence and reduce tumor growth [30, 31].

### 2.4 Tryptophan

Tryptophan metabolism plays a key role in immune suppression through the kynurenine (Kyn) pathway. The enzymes indoleamine 2,3‐dioxygenase 1 (IDO1) and tryptophan 2,3‐dioxygenase (TDO) catalyze the conversion of tryptophan into Kyn, which binds to the aryl hydrocarbon receptor (AHR) to induce regulatory T cell (Treg) expansion and suppress cytotoxic T cell function [12, 32, 60, 61]. Many cancers upregulate IDO1 and TDO, leading to tryptophan depletion and enhanced immune evasion [62, 63]. This pathway is well characterized in renal cell carcinoma and melanoma.

Several IDO1 inhibitors, such as epacadostat, have been developed to counteract this immunosuppressive mechanism [35, 64]. However, clinical trial outcomes have demonstrated inconsistent efficacy, indicating that combination approaches with immune checkpoint inhibitors (ICIs) might be required for optimal therapeutic outcomes [65, 66].

### 2.5 Serine and Methionine

Serine and methionine are integral to one‐carbon metabolism, supporting nucleotide synthesis, methylation reactions, and antioxidant defense mechanisms [36, 37, 67]. Tumors frequently upregulate serine synthesis via phosphoglycerate dehydrogenase (PHGDH), providing critical intermediates for biosynthesis and epigenetic regulation [38, 44, 68]. Methionine metabolism influences DNA and histone methylation through its derivative, S‐adenosylmethionine (SAM), which modulates gene expression and tumor progression [39, 69, 70]. These mechanisms have been reported in hepatocellular carcinoma and triple‐negative breast cancer.

Methionine depletion strategies, including dietary restriction and inhibitors targeting methionine adenosyltransferase 2A (MAT2A), have been explored to limit cancer cell proliferation while enhancing T cell responses in the TME [40, 41, 71].

### 2.6 Other Amino Acids

In addition to the major amino acids discussed above, several other amino acids contribute to tumor metabolism and immune regulation. For instance, c*ysteine* is critical for glutathione synthesis, protecting cells from oxidative stress [72]. Cysteine depletion in the TME can impair T cell function and promote tumor progression [73]. *Asparagine* supports protein synthesis and metabolic adaptation in cancer cells [74, 75]. Tumors with high asparagine demand may be targeted with asparaginase therapy [76, 77]. *Branched‐chain amino acids (BCAAs)* such as leucine, isoleucine, and valine regulate mTORC1 signaling and energy metabolism, influencing tumor cell growth and immune responses [78, 79, 80]. *Histidine* metabolism affects polyamine synthesis and histamine production, with potential implications for inflammation and tumorigenesis [81, 82]. *Proline* contributes to collagen synthesis and redox homeostasis, supporting tumor growth and microenvironment remodeling [83, 84]. These metabolic features are observed across different tumor types, including prostate cancer, endometrial cancer, and breast cancer.

Collectively, these pathways illustrate how tumor cells exploit amino acid metabolism to simultaneously sustain biosynthesis and modulate immune responses. Understanding the broader landscape of amino acid metabolism in cancer offers new therapeutic choices for selectively targeting metabolic vulnerabilities while preserving immune function.

## 3 Lipid Metabolism in Cancer

Lipid metabolism in cancer is not only a fuel or membrane supply pathway but also a signaling platform that drives tumor progression and immune evasion. Tumor cells increase lipid uptake and lipogenesis while reshaping FAO, cholesterol metabolism, lipid mediator synthesis, and lipid peroxidation programs. These changes can impair DC cross‐presentation, reinforce suppressive myeloid states, and promote CD8^+^ T cell dysfunction, thereby reducing response to immunotherapy.

### 3.1 Overview of Lipid Metabolic Reprogramming in Cancer

**FIGURE 2:**
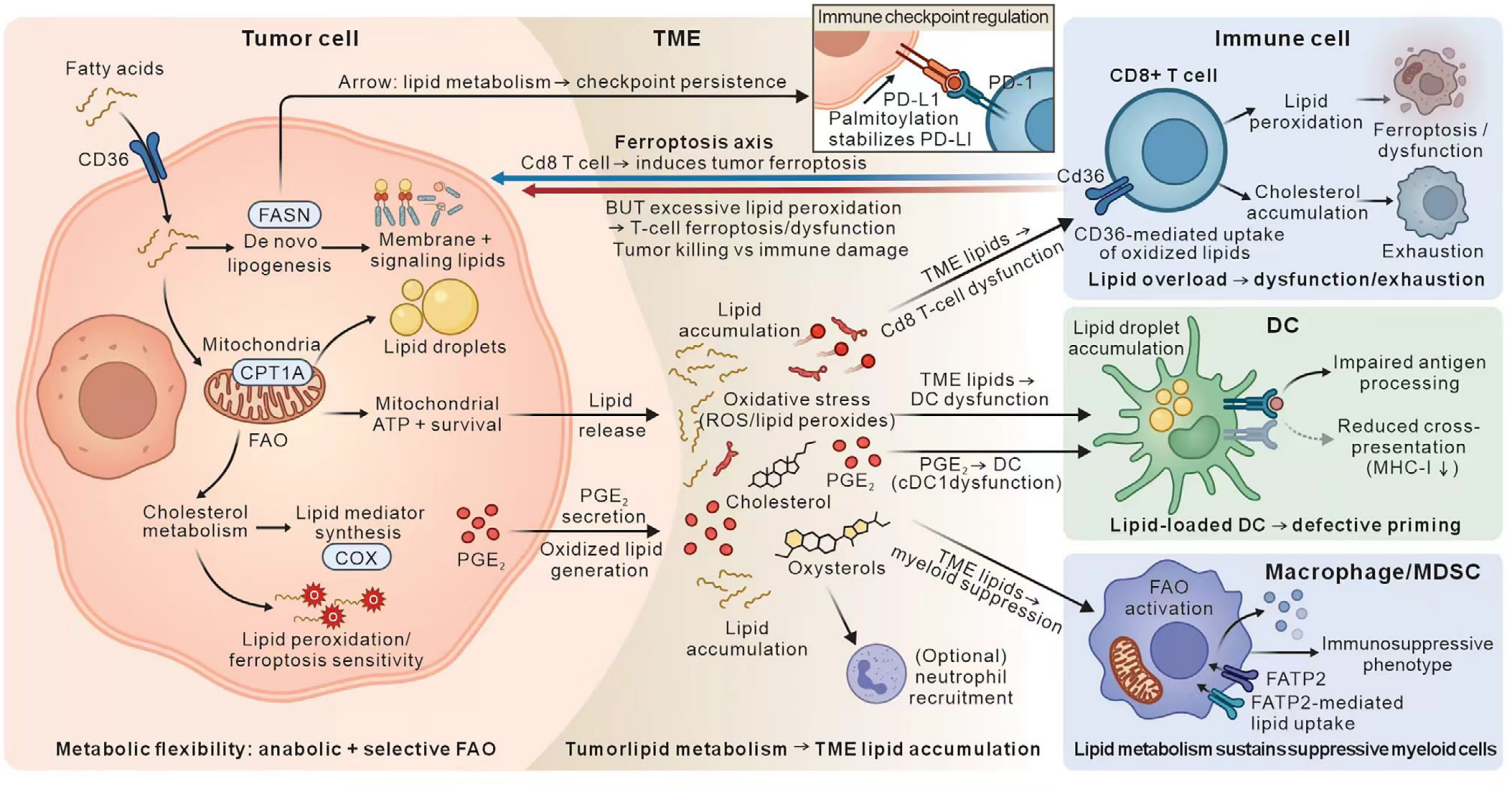
Tumor lipid metabolic reprogramming links lipid accumulation, immune suppression, and ferroptosis sensitivity. Tumor cells increase CD36‐mediated fatty acid uptake, FASN‐driven de novo lipogenesis, CPT1A‐dependent FAO, cholesterol metabolism, COX‐PGE_2_ lipid mediator synthesis, and lipid peroxidation/ferroptosis sensitivity. These pathways generate lipid droplets, membrane and signaling lipids, mitochondrial ATP, PGE_2_, oxidized lipids, cholesterol/oxysterols, and TME lipid accumulation, thereby promoting PD‐L1 palmitoylation and checkpoint persistence, impairing DC antigen processing and cross‐presentation, sustaining macrophage/MDSC immunosuppression, and inducing CD8^+^ T cell dysfunction or ferroptosis. The ferroptosis axis has a dual effect: CD8^+^ T cells can induce tumor ferroptosis, whereas excessive lipid peroxidation may damage effector T cells. ATP, adenosine triphosphate; CD8, cluster of differentiation 8; CD36, cluster of differentiation 36; cDC1, type 1 conventional dendritic cell; COX, cyclooxygenase; CPT1A, carnitine palmitoyltransferase 1A; DC, dendritic cell; FAO, fatty acid oxidation; FASN, fatty acid synthase; FATP2, fatty acid transport protein 2; MDSC, myeloid‐derived suppressor cell; MHC‐I, major histocompatibility complex class I; PD‐1, programmed cell death protein 1; PD‐L1, programmed death ligand 1; PGE_2_, prostaglandin E_2_; ROS, reactive oxygen species; TME, tumor microenvironment.

Lipids support cancer growth through multiple layers: structural membrane biogenesis, energy storage, mitochondrial ATP generation, and production of bioactive mediators. In many tumors, the lipid program is simultaneously anabolic (lipogenesis and lipid storage) and selective‐catabolic (FAO in specific stress or resistance contexts), reflecting metabolic flexibility rather than a single “lipid‐high” state [85]. Importantly, lipid remodeling does not remain tumor‐intrinsic; it changes the TME lipid composition, redox status, and paracrine mediator landscape, which together reprogram immune cell function and antitumor immune surveillance [86, 87, 88, 89] (Figure 2).

### 3.2 Lipid Uptake and Storage

#### 3.2.1 Lipid Uptake as a Growth and Immune‐Evasion Mechanism

Tumors frequently increase lipid uptake to support proliferation and survival under nutrient limitation. The fatty acid receptor/transport node CD36 has been linked to metastasis initiation and aggressive behavior in some tumor contexts, supporting the concept that exogenous lipid acquisition can promote a metastatic phenotype [90]. Lipid uptake is also relevant for immune cells: in the TME, high lipid availability and oxidative lipid species can impose functional constraints on CD8^+^ T cells and can provide survival advantages to suppressive subsets (discussed below) [18, 91, 92, 93].

#### 3.2.2 De Novo Lipogenesis and Its Immune Consequences

De novo fatty acid synthesis supports membrane expansion and generates signaling lipids that sustain oncogenic pathways; this theme is well established in cancer biology [94]. Beyond growth support, tumor lipogenesis may indirectly promote immune evasion by shaping membrane protein stability and antigen presentation competence. For example, inhibition of FASN was reported to reduce MHC‐I degradation and synergize with PD‐L1 blockade in hepatocellular carcinoma models, consistent with a lipid‐dependent control of antigen presentation and T cell sensitivity [95]. Lipid synthesis is also relevant for lymphocyte fate decisions: de novo fatty acid synthesis can influence T cell differentiation programs, making it a potential “double‐edged” intervention point where the target compartment (tumor vs. immune cell) becomes critical [96].

#### 3.2.3 Lipid Storage and Lipid Droplets as Functional Organelles

Lipid droplets act as reservoirs for neutral lipids and can buffer metabolic stress. In antigen‐presenting cells, abnormal lipid droplet accumulation correlates with impaired antigen handling; in tumor‐bearing hosts, DC lipid loading is associated with decreased functional cross‐presentation and weakened initiation of antitumor CD8^+^ T cell responses [86, 87, 88]. In macrophages, lipid droplet‐linked FA metabolism supports suppressive polarization and immune‐evasive tissue remodeling. Mechanistic work has shown that lipid droplet‐dependent FA metabolism can sustain immunosuppressive tumor‐associated macrophage (TAM) phenotypes [97, 98].

### 3.3 Fatty Acid Oxidation and Lipid Catabolism

#### 3.3.1 Tumor‐Intrinsic FAO as Adaptive Resistance to Cytotoxic Immunity

Although many cancers emphasize anabolic lipid pathways, tumor‐intrinsic FAO can be induced as an adaptive resistance program under immune attack. A *PNAS* study reported that cytotoxic stress from killer T cells induces CPT1A upregulation and FAO activation in cancer cells, and that repression of CPT1A activity/expression increases susceptibility to cytotoxic T lymphocyte killing, indicating immune‐driven selection for FAO‐based resistance [99]. This is clinically relevant because it suggests that FAO inhibition may function as an “immunotherapy sensitizer” in selected contexts rather than a generic cytotoxic approach.

#### 3.3.2 FAO in Suppressive Myeloid Cells

FAO is also a core metabolic support for immunosuppressive myeloid populations. Inhibition of FAO reduces immunosuppressive functions of MDSCs and can enhance anticancer therapies in preclinical models [100]. Beyond FAO itself, lipid uptake machinery can be rate‐limiting: a *Nature* study showed that fatty acid transport protein 2 (FATP2) reprograms neutrophils in cancer, supporting an immunosuppressive phenotype through lipid metabolic remodeling [101]. In TAMs, both lipid accumulation and active lipid metabolism are required for differentiation and activation, reinforcing the concept that lipid handling is not a passive marker but a functional dependency of suppressive myeloid states [97, 98].

#### 3.3.3 FA Metabolism in Effector T Cells as a Metabolic Checkpoint

Effector lymphocytes must balance lipid use with redox control. In the TME, lipid overload and oxidative lipid uptake can impair effector activity, while certain interventions that restore productive lipid utilization and limit peroxidative damage may strengthen effector persistence [18, 91, 92, 93]. A recent *Cell Metabolism* study reported that acetyl‐CoA carboxylase (ACC) can obstruct CD8^+^ T cell lipid utilization in the TME, and that restraining this node rewires CD8^+^ TIL metabolism toward improved tumor control, highlighting a lipid‐utilization checkpoint within TILs [102]. These data support that lipid metabolism can be targeted not only in tumor cells but also as a direct lever of T cell function.

### 3.4 Lipid Peroxidation and Ferroptosis

#### 3.4.1 Ferroptosis as a Tumor Vulnerability Shaped by Immunity

Ferroptosis is a regulated form of cell death driven by iron‐dependent lipid peroxidation, initially defined as distinct from apoptosis and necrosis [103]. In cancer immunity, ferroptosis has dual roles: it can be induced in tumor cells as a therapeutic vulnerability, but it can also damage immune cells if antioxidant defenses are overwhelmed.

A *Nature* study demonstrated that CD8^+^ T cells regulate tumor ferroptosis during cancer immunotherapy, linking adaptive immunity to lipid peroxidation‐driven tumor killing [104]. This provides a mechanistic bridge between immune effector activity and lipid redox biology, and supports ferroptosis pathways as potential synergy points with immunotherapy.

#### 3.4.2 Lipid Peroxidation as a Mechanism of T cell Dysfunction

Oxidative lipid stress can also suppress immunity. Uptake of oxidized lipids via the scavenger receptor *CD36* was shown to promote lipid peroxidation and dysfunction in intratumoral CD8^+^ T cells [18]. Complementing this, CD36‐mediated ferroptosis was reported to dampen intratumoral CD8^+^ T cell effector function and impair antitumor activity, supporting lipid peroxidation as a direct driver of T cell failure in lipid‐rich TMEs [91]. These findings imply that tumor therapies that increase oxidative lipid stress must consider the “collateral” risk on effector lymphocytes.

#### 3.4.3 Regulatory Nodes of Ferroptosis Susceptibility

Multiple enzymes shape ferroptosis sensitivity by controlling phospholipid composition and peroxidation potential. ACSL4 was shown to dictate ferroptosis sensitivity by shaping cellular lipid composition [105]. Pharmacologic LPCAT3 inhibitors remodel polyunsaturated phospholipid content and protect from ferroptosis, indicating that lipid remodeling can tune ferroptosis thresholds [106]. In immune biology, T cell lipid peroxidation can drive ferroptosis and prevent protective immunity in infection models, illustrating that immune cell ferroptosis is biologically plausible and functionally meaningful [107]. Together, ferroptosis should be framed in cancer as a tumor‐killing mechanism that requires protection of effector immune cells.

### 3.5 Cholesterol Metabolism and Oxysterol Signaling

Cholesterol is essential for membrane organization and receptor signaling, but dysregulated cholesterol handling contributes to immune dysfunction. A *Cell Metabolism* study reported that cholesterol induces CD8^+^ T cell exhaustion in the TME, associated with impaired antitumor function [92]. Conversely, modulating cholesterol metabolism can enhance T cell performance; a *Nature* paper demonstrated that altering cholesterol handling can potentiate the antitumor response of CD8^+^ T cells [93]. Cholesterol regulators can also affect tumor antigen presentation and immunotherapy sensitivity: inhibition of PCSK9 was shown to potentiate immune checkpoint therapy, consistent with cholesterol‐linked control of immune recognition pathways [108].

Oxysterols provide additional immunoregulatory cues. The oxysterol‐CXCR2 axis was implicated in recruiting tumor‐promoting neutrophils, illustrating how cholesterol‐derived metabolites can rewire immune trafficking and tumor‐promoting inflammation [109].

### 3.6 Lipid‐Derived Mediators and Immune Orchestration

#### 3.6.1 COX‐PGE_2_ Axis as an Immune‐Evasion Program

Cyclooxygenase (COX)‐dependent prostaglandin synthesis is a major lipid mediator pathway in cancer. A *Cell* study demonstrated COX‐dependent tumor growth through evasion of immunity, supporting prostaglandin pathways as bona fide immune escape mechanisms rather than only inflammatory byproducts [110]. Newer mechanistic work has shown that tumor‐derived PGE_2_ can program cDC1 dysfunction and impair intratumoral orchestration of anticancer T cell responses, positioning PGE_2_ as a direct regulator of antigen‐presentation quality and T cell recruitment/priming [111]. The importance of DC‐NK cooperation is also highlighted by evidence that NK cells stimulate recruitment of cDC1 into tumors to support immune control, implying that lipid mediators disrupting this axis can have broad immunosuppressive impact [112].

#### 3.6.2 PGE_2_ Interfaces With Checkpoint‐Like T cell Failure

Prostaglandin E_2_ and PD‐1 signaling were reported to coordinately impair CTL function and survival in chronic infection models, reinforcing that lipid mediators can synergize with inhibitory receptor pathways to drive dysfunction states [113]. This conceptual link is relevant to tumors, where PD‐1‐dominant exhaustion programs coexist with high prostaglandin level.

#### 3.6.3 Leukotrienes and Sphingolipid Signaling

Leukotrienes are key regulators of immune trafficking. In the *Journal of Immunology*, expression of leukotriene B4 receptor‐1 (BLT1) on CD8^+^ T cells was required for migration into tumors and effective antitumor immunity, illustrating how lipid mediators integrate with T cell localization, not only cytotoxic function [114]. Sphingolipid signaling can impose a metabolic brake on T cells: a *Cell Reports* study reported that sphingosine‐1‐phosphate can metabolically program T cells to limit antitumor activity [115]. Tumor‐intrinsic sphingolipid enzymes are also linked to checkpoint regulation; *Cellular & Molecular Immunology* reported that sphingosine kinase 1 promotes tumor immune evasion by regulating an MTA3‐PD‐L1 axis [116]. In addition, altered sphingomyelin metabolism can contribute to immune escape; *Cancer Research* reported a role for acid sphingomyelinase in shaping tumor immune escape programs [117].

### 3.7 Lipid Metabolism and Immune Checkpoint Regulation

Immune checkpoints can be regulated by lipid‐dependent protein modifications and membrane trafficking. In *Nature Biomedical Engineering*, inhibiting PD‐L1 palmitoylation was shown to enhance T cell immune responses against tumors, implying that lipid post‐translational modifications stabilize checkpoint function at the protein level [118]. Tumor lipogenesis can also affect antigen presentation and checkpoint sensitivity; as noted above, FASN inhibition reduced MHC‐I degradation and synergized with PD‐L1 blockade in hepatocellular carcinoma models, connecting lipid synthesis to immune visibility and therapy response [95]. These findings support a practical model: lipid metabolism regulates immune escape not only through soluble mediators (PGE_2_, oxysterols) but also by controlling surface checkpoint persistence and antigen presentation competence.

## 4 Summary

Lipid metabolism in cancer should be discussed as a coupled tumor–immune system: tumors increase lipid uptake/lipogenesis and selectively deploy FAO and cholesterol programs that shape immune infiltration, antigen presentation, and effector exhaustion (Table 2). DC lipid loading and PGE_2_‐driven cDC1 dysfunction weaken priming; suppressive myeloid cells depend on lipid uptake/FAO; and oxidative lipids and ferroptosis‐linked stress drive CD8 T cell dysfunction. Therapeutically, lipid pathways offer multiple intervention nodes, but the net effect depends on which compartment (tumor vs. immune) is dominantly modulated.

**TABLE 2:** Effect of major lipid metabolism pathways and their effects on cancer immunity.

Lipid pathway	Representative mechanism	Dominant immune effect in TME	Key refs
Lipid uptake and storage	Lipid accumulation in DCs and immune cells; oxidized lipids interfere with processing	Impaired cross‐presentation and weakened CD8 priming	[86, 87, 88]
Myeloid lipid programs	FATP2‐driven neutrophil reprogramming; FAO dependence of MDSCs/TAMs	Reinforced immunosuppressive myeloid phenotypes	[97, 98, 100, 101]
Oxidized lipid stress	CD36‐driven oxidized lipid uptake and peroxidation in CD8^+^ T cells	CD8 dysfunction/exhaustion‐like failure	[18, 91]
Cholesterol remodeling	Cholesterol accumulation in CD8 TILs; cholesterol‐handling regulators	Exhaustion programs vs. improved TCR function depending on node targeted	[92, 93, 108, 109]
Eicosanoids (COX‐PGE_2_)	COX‐dependent immune evasion; PGE_2_‐driven cDC1 dysfunction	Reduced DC orchestration and weakened T cell immunity	[110, 111, 112, 113]
Leukotrienes and sphingolipids	BLT1‐dependent CD8 trafficking; S1P metabolic programming; SPHK1‐PD‐L1 axis	Altered infiltration and reduced effector activity; checkpoint upregulation	[114, 115, 116]
Ferroptosis	Immune‐driven tumor ferroptosis; T cell ferroptosis risk under lipid peroxidation	Tumor killing potential but immune cell vulnerability	[103, 104, 105, 106, 107]
Lipid PTMs and checkpoints	PD‐L1 palmitoylation stabilizes checkpoint function	Prolonged immune inhibition; targetable checkpoint stability	[95, 118]

## 5 Impact on Immune Cells

In the TME, immune cells are exposed to nutrient competition and metabolic stress created by tumor cells and suppressive stromal or myeloid cells. This section discusses how amino acid and lipid metabolism regulate immune cell function, including T cell activation and exhaustion, DC antigen presentation, macrophage and MDSC polarization, and NK cell cytotoxicity. We first summarize the immunological effects of major amino acid pathways, then discuss lipid‐driven immune regulation, and finally integrate these changes from the view of nutrient competition and response to immune checkpoint blockade.

### 5.1 Amino Acid Metabolism and Immune Regulation

**FIGURE 3:**
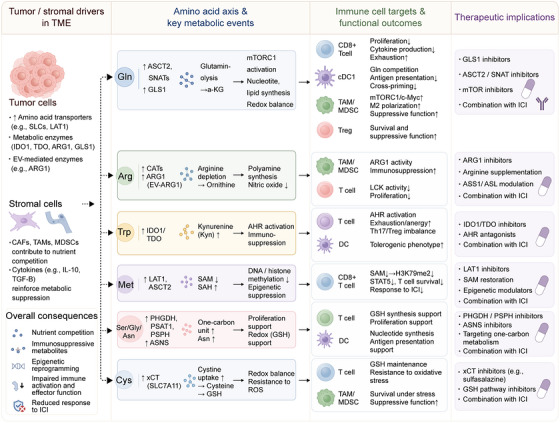
Amino acid metabolic axes shape immune cell dysfunction and therapeutic vulnerabilities in the tumor microenvironment. This figure summarizes how tumor and stromal cells remodel glutamine, arginine, tryptophan, methionine, serine/glycine/asparagine, and cystine metabolism to drive nutrient competition, immunosuppressive metabolite accumulation, epigenetic reprogramming, redox adaptation, and impaired immune activation. These metabolic events suppress CD8+ T cell proliferation and effector function, promote Treg and myeloid‐cell‐mediated immunosuppression, impair dendritic cell antigen presentation, and contribute to reduced responses to immune checkpoint inhibitors. Therapeutic strategies targeting these axes include inhibitors of GLS1, ASCT2/SNAT, ARG1, IDO1/TDO, AHR, LAT1, PHGDH/PSPH, ASNS, xCT, and GSH‐related pathways, as well as combination approaches with immune checkpoint blockade. AHR, aryl hydrocarbon receptor; ARG1, arginase 1; ASCT2, alanine‐serine‐cysteine transporter 2; CATs, cationic amino acid transporters; EV, extracellular vesicle; GSH, glutathione; IDO1, indoleamine 2,3‐dioxygenase 1; Kyn, kynurenine; LAT1, l‐type amino acid transporter 1; PHGDH, phosphoglycerate dehydrogenase; PSAT1, phosphoserine aminotransferase 1; PSPH, phosphoserine phosphatase; SAH, S‐adenosylhomocysteine; SAM, S‐adenosylmethionine; SNATs, sodium‐coupled neutral amino acid transporters; TAMs, tumor‐associated macrophages; TDO, tryptophan 2,3‐dioxygenase; Tregs, regulatory T cells; xCT/SLC7A11, cystine/glutamate antiporter.

In solid tumors, immune cells must compete with malignant and stromal cells for limited nutrients; among these, amino acids are particularly relevant because they simultaneously support biosynthesis and provide upstream signaling cues for activation programs (e.g., amino acid transporter induction, mTORC1 engagement, and stress‐response wiring) [119, 120]. Experimental models show that changing amino acid access can shift the balance between effector differentiation and dysfunctional/exhausted states, influencing response to checkpoint blockade [121, 122, 123]. Clinically, the complexity is reflected by mixed outcomes of single‐pathway targeting (e.g., IDO1 inhibition), suggesting that amino acid immunoregulation is frequently redundant and context‐dependent [122] (Figure 3, Table 3).

**TABLE 3:** Effects of major amino acid metabolism in TME on immune cells.

Amino acids	Immune cells	Transporter	Influence
Gln	T cells	SLC38A2	Gln inhibits Treg proliferation, promotes CD8^+^ T infiltration, and increases effector molecule expression [52, 124, 125].
	B cells		Gln promotes the differentiation of AtMBs by upregulatingα‐KG [126].
	TAMs		Gln promoted the polarization of M2‐type macrophages by activating the mTORC1/c‐Myc signaling pathway [15].
	MDSCs		Under Gln restriction conditions, it increased the expression of tumor necrosis factor (TNF) and promoted the conversion of MDSCs to proinflammatory TAMs [127].
	DCs		Gln regulates cDC1 function via the FLCN‐TFEB signaling axis, and loss of FLCN results in impaired cDC1 antigen delivery capacity [124].
Trp	T cells	SLC7A5	Trp deficiency suppresses the activity of Tregs and simultaneously upregulates the activity of Teff cells [61, 128].
Arg	TAMs		Reduction of Arg and its metabolites suppresses the polarization of M2 macrophages [129].
	T cells		Arg is able to reduce the inhibitory function of Treg cells by activating the mTOR pathway [130].
Met	T cells	SLC43A2	The increase of SAM in tumor suppresses T cell activity, but the creation of an acidic environment helps the T to maintain cell stemness. SAM in T cells enhances T cell activity by promoting H3K79me2 [131].
Leu	T cells		The increase of Leu levels, promoting the translocation of mTORC1 to lysosomes, could enhance mTORC activity [78].
	NEs		Leu upregulates histone H3K27 acetylation and enhances the antigenic effects of neutrophils [132].
	B cells		Leu promotes immune evasion through the upregulation of TGF‐b1 in B cells [133].
Asn	T cells		Asn binds to and activates LCK, which promotes T cell activation by enhancing the TCR signaling pathway [74].
	MDSCs	SLC25A22	Asn promotes signal transduction in the ERK/ETS 2 pathway, enabling a significant upregulation of the chemokine CXCL1 transcription and attracting the aggregation of MDSCs [134].
Asp	TAMs		The Asp metabolite spermidine upregulates the expression of HIF‐1α, driving the reprogramming of glycolysis in TAM [14, 135].
Glu	TAMs/T cells/granulocyte	SLC25A13	SLC25A13 maintains the redox balance of immune cells by regulating glutathione synthesis [136, 137].
Tau	T cells	SLC6A 6	Tau enhances T cell proliferation and function by stimulating the PLC γ 1‐mediated calcium and MAPK signaling pathways. In Tau deficiency, the PERK‐JAK1‐STAT3 pathway‐mediated up‐regulation of ATF 4 expression inhibited immune cell proliferation [138, 139, 140].
Ser	T cells		Ser deficiency leads to the accumulation of mtDNA in mitochondria, and mtDNA activates the cGAS‐STING 1 signaling pathway to upregulate the secretion of type I interferon (IFN), thus promoting the infiltration of T cells in tumors [141].
Cys	T cells	Slc7a11	Cys deficiency leads to oxidative stress and lipid peroxide accumulation in T cells and induces iron death in T cells [73].
Lys	T cells	SLC7A2	The Lys metabolite, crotonyl‐CoA, reduces the activation of the type I interferon signaling pathway and inhibits T cell activity [142].

### 5.2 Glutamine Axis: Tumor–Immune Divergence and Therapeutic Leverage

Glutamine supports both tumor growth and immune activation; however, key studies indicate a potentially exploitable asymmetry in metabolic plasticity. In a *Science* study using a glutamine antagonist strategy, glutamine blockade suppressed tumor oxidative/glycolytic programs and reduced features of an immunosuppressive microenvironment (including metabolic stressors such as acidosis/hypoxia surrogates), while effector T cells increased oxidative metabolism and adopted a more durable activated phenotype, enabling potent antitumor activity. This “divergent adaptation” model provides a mechanistic rationale for glutamine‐targeted therapy as an immunometabolic checkpoint rather than only a cytostatic approach [121].

At the immune cell level, glutamine uptake and sensing are tied to activation circuitry. Inflammatory Th1/Th17 differentiation requires the glutamine transporter ASCT2/SLC1A5 and intact mTORC1 activation, establishing a direct transporter‐to‐signaling linkage in T cells [119]. Beyond T cells, glutamine availability also shapes myeloid compartments. A *Journal of Clinical Investigation* study reported that targeting glutamine metabolism can enhance tumor‐specific immunity by modulating suppressive myeloid cells, supporting the idea that glutamine interventions may relieve myeloid‐driven resistance features [143]. A *Molecular Cell* paper further showed that inhibiting glutamine utilization may synergize with immune checkpoint blockade, emphasizing combination logic rather than monotherapy framing [144].

Recent work also highlights antigen‐presenting cells as glutamine‐sensitive nodes. A *Nature* study demonstrated that SLC38A2 and glutamine signaling in cDC1s influence antitumor immunity, reinforcing that amino acid control can operate upstream of T cell priming quality, not only at the effector phase [124]. In more translational settings, myeloid‐rich tumors may be particularly sensitive to glutamine antagonism‐based reprogramming strategies, with TAM phenotypes being modulated toward immune‐permissive states in preclinical contexts [145]. Collectively, the glutamine axis is best conceptualized as a multi‐compartment regulator.

### 5.3 Arginine Axis: ARG1‐Mediated Deprivation, EV Transfer, and Downstream Polyamine Biology

Arginine is classically linked to T cell proliferation and TCR signaling competence. Mechanistic studies showed that macrophage arginine consumption can reduce CD3ζ chain expression and impair T cell proliferation/cytokine production, connecting extracellular arginine depletion to antigen receptor signaling integrity [146]. In tumors, myeloid populations expressing high arginase activity can rapidly deplete arginine, producing functional T cell defects and supporting immune escape [147].

A parallel line of evidence emphasizes systemic and intercellular delivery mechanisms: small extracellular vesicles containing arginase‐1 can suppress T cell responses and promote tumor growth; in vivo, these vesicles can reach draining lymph nodes and affect antigen‐specific activation, suggesting that arginine deprivation may be distributed beyond the immediate tumor niche [148].

Therapeutically, pharmacologic arginase inhibition can remodel the microenvironment. In a previous study, arginase inhibition (CB‐1158) blocked myeloid‐mediated immune suppression, increased CD8/NK infiltration signatures, and improved tumor control including in combinations [30]. More broadly, arginine availability also has cell‐intrinsic consequences for T cell fate: elevating intracellular arginine levels shifts activated T cells toward oxidative metabolism and promotes memory‐like features with improved antitumor activity in vivo [146]. Thus, strategies that restore arginine bioavailability may simultaneously correct signaling defects (CD3ζ), improve metabolic fitness, and enhance persistence‐like programs [149].

Importantly, arginine metabolism connects to polyamine synthesis. Polyamine elevation contributes to tumor immunosuppression and can be therapeutically targeted. Polyamine‐blocking therapy has been shown to reverse immunosuppression within the TME and exert antitumor immune effects [150]. Additional work indicates that polyamine modulation can decrease survival of immunosuppressive myeloid subsets and improve PD‐1 blockade efficacy in resistant tumor models [151]. Emerging mechanistic studies further suggest that polyamines can regulate adaptive antitumor immunity through regulatory T cell functional programming, highlighting that downstream arginine pathways can be immunologically active beyond simple nutrient depletion [152].

### 5.4 Tryptophan–Kynurenine Axis: IDO/TDO, AHR Signaling, and Clinical Translation Gap

Tryptophan catabolism is one of the most studied immune escape pathways. A seminal *Nature Medicine* paper provided evidence for a tumor immune resistance mechanism based on tryptophan degradation by IDO, supporting both tryptophan deprivation and kynurenine accumulation as immunoregulatory forces [62]. Earlier immunology work in pregnancy tolerance showed that inhibiting IDO‐mediated tryptophan catabolism can trigger T cell‐driven rejection, establishing a strong biological precedent for tryptophan catabolism as a tolerance mechanism [153]. A synthesis framework is provided by Mellor and Munn, emphasizing IDO expression (notably in DCs) as a tolerance‐promoting program with relevance to tumors [85].

A key mechanistic advance was identifying kynurenine as an endogenous ligand for the aryl hydrocarbon receptor (AHR). In *Nature*, Opitz et al. reported that TDO‐derived kynurenine activates AHR and can suppress antitumor immune responses, linking tumor‐intrinsic tryptophan catabolism to immune evasion via a defined receptor pathway [63]. In parallel, immune cell‐focused work showed that kynurenine‐AHR interaction can promote regulatory T cell generation, providing a plausible cellular mechanism for immunosuppression [154]. Tumor studies further reported that kynurenine can drive PD‐1 upregulation in CD8^+^ T cells through transcellular delivery and AHR activation, directly coupling metabolic by‐products to checkpoint receptor expression programs [155].

Therapeutically, targeting downstream signaling can be effective in models: blockade of the AHR restricted a Treg–macrophage suppressive axis induced by l‐kynurenine and enhanced anti‐PD‐1 activity, supporting AHR as a convergence node for IDO/TDO biology [33]. However, clinical translation has been challenging. A phase 3 study combining the IDO1 inhibitor epacadostat with pembrolizumab in advanced melanoma did not show clinical benefit over pembrolizumab alone, underscoring that pathway redundancy, pharmacodynamics, and patient stratification are major limitations for single‐enzyme targeting [122]. Therefore, it is reasonable to position the tryptophan axis as mechanistically strong but clinically complex.

### 5.5 Other Amino Acids in Immune Regulation

Beyond the three canonical axes, several other amino acids act through epigenetic, redox, and signaling mechanisms.

Methionine availability can be shaped by transporter‐level competition. A *Nature* paper described how cancer cells can alter T cell methionine metabolism and histone methylation through methionine transport competition, impairing effector function via epigenetic constraints [131]. More recent work indicates that methionine limitation can progressively upregulate PD‐1 expression in CD4 T cells through epigenetic and AMPK‐linked mechanisms, thereby weakening antitumor immunity in a nutrient‐dependent manner [156]. In parallel, early methionine availability can influence exhaustion trajectories, consistent with methionine being a timing‐sensitive determinant of fate decisions [157]. Dietary or metabolic manipulation also affects tumor‐side checkpoint expression: methionine‐derived SAM can promote m^6^A‐related control of immune checkpoint transcripts (including PD‐L1 and VISTA), and methionine restriction can enhance CD8 infiltration and cytotoxicity in models, providing tumor‐intrinsic and immune‐extrinsic angles for intervention [39].

Serine/glycine pathways are closely coupled to one‐carbon metabolism and effector expansion. Foundational work demonstrated serine as an essential metabolite for effector T cell expansion, linking amino acid availability to proliferative and functional output [123]. A more recent *Cell Metabolism* study further investigated a serine/glycine‐free diet approach, showing enhanced cytotoxic T cell accumulation and a rationale for combination with PD‐1/PD‐L1 blockade, while also identifying adaptive immune‐evasion features (e.g., PD‐L1 modification pathways) that may arise under dietary pressure [158].

Cystine/cysteine availability touches redox homeostasis and ferroptosis‐linked tumor killing. A *Nature* study showed that immunotherapy‐activated CD8^+^ T cells can promote tumor ferroptosis and that the cystine transporter axis is involved, connecting immune effector signals (including IFN‐γ context) to metabolic vulnerability in tumor cells [104]. Complementary work indicates that cystine deprivation can drive dysfunction programs in tumor‐infiltrating CD8^+^ T cells via redox imbalance and lipid‐peroxidation stress, implying that manipulating cystine must consider both tumor sensitization and T cell fitness balance [73].

Finally, some “non‐essential” amino acids can act in a signaling‐like manner rather than as metabolic substrates. For example, asparagine has been reported to potentiate CD8 T cell activation and antitumor responses by binding and modulating LCK phosphorylation dynamics, positioning LCK as a natural “asparagine sufficiency” sensor [74].

### 5.6 Lipid Metabolism and Immune Regulation

**FIGURE 4:**
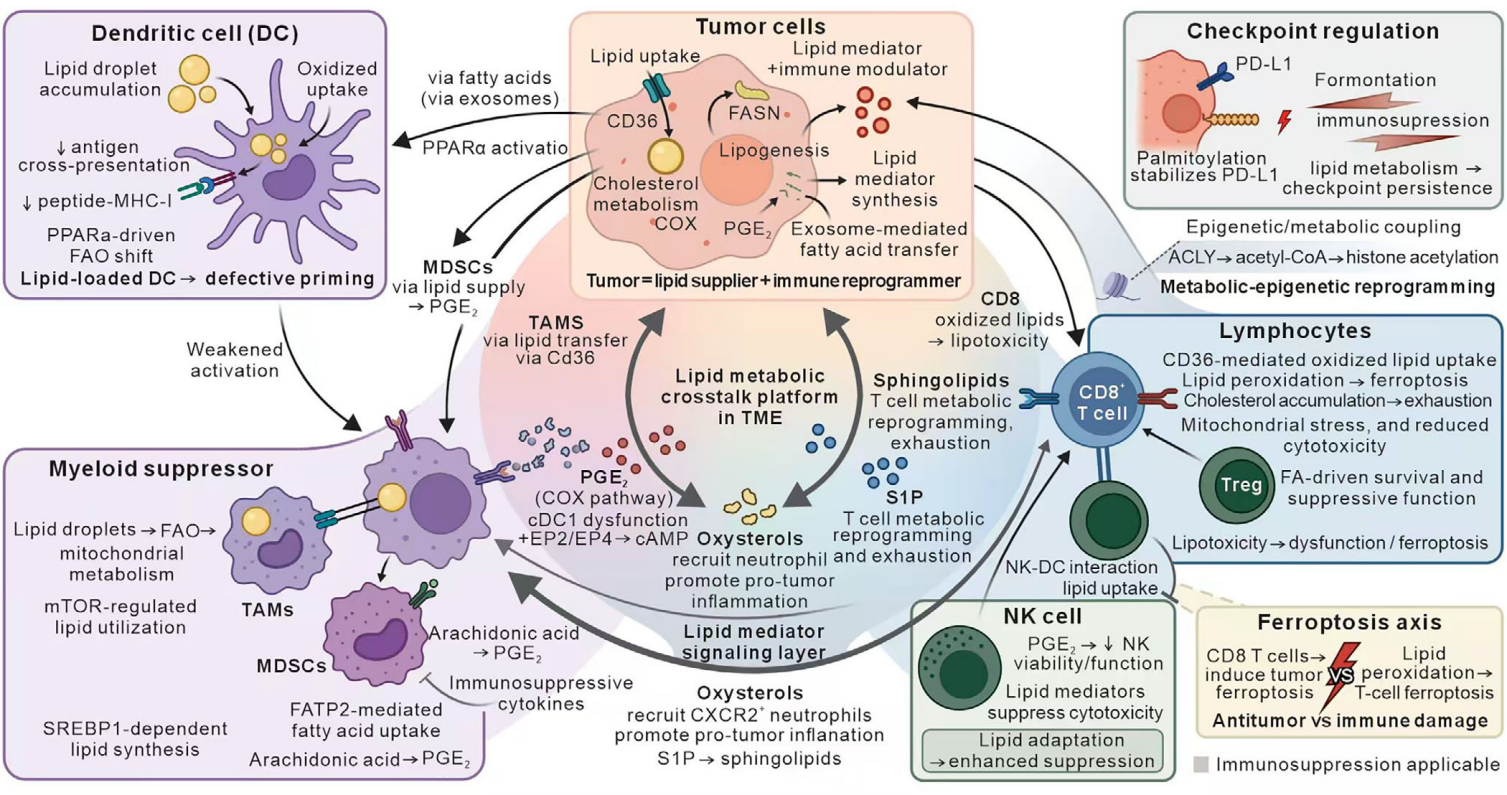
Lipid metabolism functions as a multidirectional crosstalk platform between tumor cells and immune cells. Tumor cells act as lipid suppliers and immune reprogrammers through lipid uptake, lipogenesis, cholesterol metabolism, COX‐PGE_2_ signaling, lipid mediator synthesis, and exosome‐mediated fatty acid transfer. These lipid signals induce lipid‐loaded DCs with defective priming, support TAM/MDSC lipid utilization and immunosuppressive cytokine production, promote lymphocyte lipotoxicity, cholesterol‐associated exhaustion, and ferroptosis, suppress NK cell cytotoxicity, and regulate PD‐L1 stability and metabolic–epigenetic reprogramming. The central signaling layer highlights PGE_2_–EP2/EP4–cAMP signaling, oxysterol/CXCR2‐mediated neutrophil recruitment, S1P/sphingolipid‐driven T cell reprogramming, and the balance between tumor ferroptosis and immune cell damage. ACLY, ATP citrate lyase; acetyl‐CoA, acetyl coenzyme A; cAMP, cyclic adenosine monophosphate; CXCR2, C‐X‐C motif chemokine receptor 2; EP2/EP4, prostaglandin E receptor 2/4; FA, fatty acid; mTOR, mechanistic target of rapamycin; NK, natural killer; PPARα, peroxisome proliferator‐activated receptor alpha; S1P, sphingosine‐1‐phosphate; SREBP1, sterol regulatory element‐binding protein 1; TAMs, tumor‐associated macrophages; Treg, regulatory T cell.

Lipid metabolic remodeling in tumors is not only associated with energy storage but also plays an important role in regulating immune responses in the TME [159]. In contrast to amino acids, which primarily function as metabolic checkpoints through deprivation or catabolism, lipids exert their immunoregulatory effects through simultaneous modulation of membrane composition, bioactive mediator production, and oxidative stress states. These properties allow lipid metabolism to influence immune cell behavior at multiple levels, including antigen presentation, lineage differentiation, effector function, and survival (Figure 4).

### 5.7 Lipid Uptake and Distribution: Shaping Antigen Presentation and Immune Cell Fitness

In the TME, abnormal lipid abundance and oxidation change antigen presentation and immune cell fitness at the same time. A classical and well‐supported example is DC lipid loading: tumor‐bearing hosts develop DCs with excessive triglycerides, which is linked to impaired priming capacity and reduced antitumor immunity [86]. Oxidative lipid species add another layer‐oxidized lipids can directly interfere with cross‐presentation by reducing surface peptide–MHC‐I, and “oxidatively truncated” lipids accumulating inside DC lipid bodies have been shown to block cross‐presentation in cancer settings [87, 88]. Tumors can also deliver fatty acids as a communication signal: tumor‐derived exosomes carrying fatty acids drive lipid droplet biogenesis and a shift toward FAO in DCs via PPARα signaling, culminating in dysfunctional DC states that support immune evasion [89].

### 5.8 Myeloid Lipid Metabolism: FAO‐dependent Immunosuppressive Programming

For immunosuppressive myeloid compartments, fatty acid uptake is not passive; it is actively wired. PMN‐MDSCs upregulate fatty acid transport machinery (notably FATP2/SLC27A2), which promotes arachidonic acid handling and PGE2‐linked suppressive activities [101], while tumor‐infiltrating MDSCs show increased fatty acid uptake and FAO enzyme signatures, and FAO inhibition (classically via CPT1A blockade) reduces suppressive pathways and improves T cell‐dependent tumor control [100]. In parallel, tumors themselves can become aggressive lipid scavengers: CD36‐dependent fatty acid uptake has been mechanistically linked to metastasis‐initiating capacity in epithelial tumors, suggesting that a lipid‐rich niche can be both a growth substrate and an immune‐modulating environment [90].

TAMs sit in the center of lipid‐driven immune regulation because they are both consumers of extracellular lipids and producers of immunosuppressive cues. In human and mouse tumors, macrophages are frequently lipid‐enriched, and this lipid accumulation is required for TAM differentiation/activation programs that support tumor progression [97]. Mechanistically, lipid droplets act like metabolic buffers: lipid‐droplet‐dependent long‐chain fatty acid metabolism (especially unsaturated fatty acids such as oleate) fuels mitochondrial respiration and sustains an immune‐suppressive TAM phenotype, with mTOR signaling regulating this lipid‐droplet‐to‐mitochondria axis [98].

### 5.9 Lipid Metabolic Checkpoints in Lymphocytes: Lipotoxicity and Lineage‐Dependent Adaptation

On the lymphocyte side, lipid overload becomes “lipotoxicity”: oxidized lipids (including oxLDL) taken up by CD36 drive lipid peroxidation and functional failure of intratumoral CD8^+^ T cells [18], and CD36‐driven ferroptotic stress has been directly shown to dampen CD8 effector activity in tumors (with recovery when CD36 or ferroptosis pathways are restrained) [91]. Importantly, this axis is not uniformly harmful for all T cells; intratumoral Tregs can exploit fatty acid uptake as an adaptation; CD36 supports their survival and suppressive function in tumors, consistent with the idea that the same nutrient channel can power either immunity or immune escape depending on lineage programs [160]. These findings indicate that lipid uptake does not uniformly suppress immunity but instead redistributes functional capacity across immune subsets, favoring suppressive lineages over effector populations.

Lipid metabolism in TAMs is also socially regulated by lymphocytes: intratumoral Tregs suppress CD8‐derived IFN‐γ, thereby permitting SREBP1‐driven lipid‐synthesis fitness in tumor‐promoting macrophages and stabilizing the suppressive myeloid landscape [161]. Tumor cells further push this direction using lipid transfer: in liver‐metastasis models, tumor‐derived extracellular vesicles enriched in long‐chain fatty acids are internalized by macrophage CD36, reprogramming macrophage lipid handling and ultimately suppressing local CD8 immunity; macrophage CD36 loss restores CD8 function and reduces metastatic outgrowth [162]. These observations make lipid metabolism a crosstalk platform, where tumor cells supply lipids, stromal/myeloid cells remodel them, and lymphocytes pay the cost in function or benefit depending on lineage. At the same time, immune cells also need de novo lipid synthesis for some effector fates: ACC1‐dependent lipogenesis supports Th17 differentiation while favoring a relative Treg bias when blocked, highlighting that lipid “anabolism” is a fate determinant [96]. Likewise, SREBP activity is essential for effector T cell metabolic programming during activation, linking sterol/lipid synthesis to clonal expansion capacity [163]. A final layer is that lipid pathways can rewrite transcriptional states through metabolic–epigenetic coupling: ATP citrate lyase (ACLY) produces cytosolic acetyl‐CoA that feeds lipid biosynthesis and histone acetylation, illustrating how shifts in lipid/acetyl‐CoA flux can become stable gene‐expression changes in macrophage biology [164].

### 5.10 Lipid Metabolism and Immune Recognition: Checkpoints and Antigen Presentation

Tumor‐intrinsic lipid synthesis also shapes immune recognition by controlling surface interfaces such as checkpoint ligands and antigen presentation. PD‐L1 biology provides a clear example: PD‐L1 palmitoylation stabilizes the protein by limiting its degradation, and inhibiting this lipid modification decreases PD‐L1 persistence and enhances T cell‐mediated antitumor responses in vivo, giving a mechanistic link between fatty acid availability, palmitoylation chemistry, and immune escape [118, 165, 166]. In a related direction, FASN inhibition in hepatocellular carcinoma increases MHC‐I protein levels by suppressing palmitoylation‐associated lysosomal degradation, strengthening CD8‐mediated killing and showing synergy with PD‐L1 blockade in vivo [95]. Cholesterol metabolism adds another immune‐regulatory axis. Tumor tissues can be cholesterol‐enriched, and cholesterol accumulation in tumor‐infiltrating CD8^+^ T cells correlates with higher inhibitory receptor expression; experimentally, cholesterol can induce an exhausted state through ER‐stress signaling branches, with functional restoration when cholesterol stress is reduced [92, 167]. From a membrane signaling perspective, cholesterol esterification matters: inhibiting ACAT1 (SOAT1) in CD8^+^ T cells increases membrane cholesterol, promotes TCR clustering, and potentiates antitumor responses in preclinical models [93]. Finally, tumors can exploit cholesterol‐handling pathways that are immune‐related rather than purely metabolic; for example, PCSK9 inhibition improves responses to immune checkpoint therapy through a mechanism reported to be independent of classic cholesterol‐lowering logic, emphasizing that lipid regulators can control antigen presentation and immune susceptibility via unexpected routes [108]. In the stroma‐myeloid network, oxysterols act as immunosuppressive lipid messengers: tumor‐derived oxysterols recruit CXCR2+ neutrophils in an LXR‐independent manner, favoring angiogenesis and immunosuppression and thereby supporting tumor growth [109].

### 5.11 Lipid Signaling Mediators: Coordination of Immune Cell Communication

Lipid mediators and oxidative lipid death pathways then connect metabolism to immune choreography at tissue scale. COX‐derived prostaglandin E2 (PGE2) is a well‐validated immunoregulatory lipid metabolite: tumor COX activity drives immune evasion and tumor growth by suppressing productive antitumor immunity [110, 168, 169]. At a cellular‐routing level, PGE2 weakens the NK‐cDC1 partnership that supports tumor control; tumor‐derived PGE2 impairs NK cell viability/chemokine output and disrupts cDC1 recruitment programs [112]. More recently, PGE2 has been shown to directly program a dysfunctional cDC1 state inside tumors through EP2/EP4‐cAMP signaling and loss of IRF8, disabling local orchestration of CD8 responses [111]. PGE2 also suppresses NK effector functions through EP4‐focused signaling, linking lipid mediator receptors to reduced innate cytotoxicity [170]. Because checkpoint inhibition is often applied on top of these lipid landscapes, it is relevant that PGE2 can cooperate with PD‐1 signaling to impair CTL function and survival (shown clearly in chronic immune stimulation models), offering a plausible mechanistic bridge for why COX‐PGE2‐rich tumors may resist durable T cell reinvigoration [113].

Leukotriene signaling (classically downstream of 5‐LOX/ALOX5) provides an additional migration axis: BLT1 on CD8^+^ T cells is required for efficient tumor trafficking and effective tumor control in experimental models, supporting the concept that eicosanoid gradients can decide “who enters” the TME [114]. Sphingolipids are another immunoregulatory lipid family. Intracellular S1P produced by SphK1 metabolically programs T cells via PPARγ‐linked lipid handling, and genetic/pharmacologic SphK1 inhibition improves adoptive T cell antitumor activity and cooperates with PD‐1 blockade in melanoma models [115]. Beyond T cells, S1P receptor signaling contributes to TME remodeling: S1PR3 blockade reduces T cell exhaustion and recruits pro‐inflammatory macrophages, improving CAR‐T efficacy in solid tumor models [171].

### 5.12 Lipid Peroxidation and Ferroptosis: A Bidirectional Immune‐Modulating Axis

Tumors themselves can use sphingolipid enzymes to tune checkpoint biology; SPHK1 has been linked to immune evasion through transcriptional control of PD‐L1 axis components in immunocompetent melanoma systems [116], and acid sphingomyelinase activity has been shown to promote immune evasion and tumor growth in lung cancer, connecting membrane sphingolipid remodeling to antitumor immunity failure [117]. Finally, oxidative lipid damage and ferroptosis link lipid composition to both tumor killing and immune cell fragility [172]. CD8^+^ T cells activated by immunotherapy can enhance ferroptosis‐specific lipid peroxidation in tumor cells, and IFN‐γ signaling has been implicated in sensitizing tumors through the cystine‐glutamate transport axis, making ferroptosis a real component of antitumor efficacy rather than only a cell‐culture phenotype [104, 173]. The ferroptosis field also clarifies why membrane lipid composition matters: ferroptosis is defined as an iron‐dependent, lipid‐peroxidation‐driven regulated death, and T cells themselves require antioxidant protection to avoid ferroptotic collapse, as GPX4 loss drives lipid peroxidation and ferroptosis in T cells, limiting immune protection [107]. At the biochemical entry points, ACSL4 enriches membranes with long ω‐6 polyunsaturated fatty acids and thereby dictates ferroptosis sensitivity [105], while LPCAT3 controls PUFA phospholipid remodeling and its inhibition remodels polyunsaturated phospholipids and provides partial protection from ferroptosis in human cells [106]. In NK cells, where rapid effector action is essential, FAO can support responses to both viruses and tumors, with NK activation increasing fatty acid uptake and CPT1A expression‐another reminder that lipid catabolism can be pro‐immune or pro‐suppressive depending on context and redox load [174, 175, 176]. Overall, lipid uptake, storage, mediator synthesis, and oxidative lipid fate form a connected network that continuously rewires tumor–immune crosstalk in the TME.

### 5.13 Competition for Nutrients Between Tumor and Immune Cells

Nutrient competition in the TME behaves like a metabolic immune checkpoint, because tumor and stromal cells continuously uptake key substrates and leave immune cells in a low‐fuel, high‐waste niche [177, 178]. Aside from the well‐established glucose competition between tumor and immune cells [177], amino acids and lipids are also key nutrients being competed for.

Amino acids are largely controlled by transporters that connect substrate supply to mTOR signaling, translation, and chromatin states (Table 1). In T cells, large neutral amino acid uptake via LAT1/SLC7A5 is essential for metabolic reprogramming and effector differentiation after antigen receptor engagement [120, 179]. Glutamine uptake through ASCT2/SLC1A5 supports mTORC1 activation and differentiation programs (Th1/Th17) in vivo, illustrating how amino acid transport functions as a signaling requirement [119, 180]. In tumors, these same pathways become zero‐sum: tumor cells and cross‐presenting cDC1s compete for glutamine, and glutamine availability determines cDC1 function, CD8^+^ T cell accumulation, and responsiveness to immunotherapy [121, 124]. In soft tissue sarcoma models, microenvironmental glutamine levels were required for conventional DC maintenance‐particularly cDC1‐linking glutamine scarcity to impaired antigen presentation capacity and reduced tumor immunity [181]. Interventions that alter glutamine use can re‐balance this competition: in KEAP1‐mutant lung cancer models, the glutamine antagonist prodrug DRP‐104 suppressed tumor growth and enhanced antitumor T cell responses while improving checkpoint blockade response [182]. Amino acid competition is not limited to glutamine; in hepatocellular carcinoma, SLC3A2 (CD98 heavy chain) overexpression increased cancer cell lysine uptake and reduced lysine availability for T cells, decreasing STAT3 levels and impairing T cell proliferation and effector function [183]. Arginine depletion is another established immune‐suppressive sink: in pancreatic cancer, macrophage Arg1 activity supported immune suppression via arginine catabolism, and Arg1 targeting increased CD8^+^ T cell infiltration and sensitized tumors to anti‐PD‐1 [184]. In patient material, NET‐associated ARG1 created suppressive microdomains that limited T cell proliferation, and antibody‐mediated ARG1 neutralization restored CD8^+^ function and enhanced checkpoint activity in humanized systems [185]. Finally, tryptophan catabolism shifts competition into signaling metabolites: in IDO/TDO‐high tumors, the kynurenine–AHR axis drove a Treg–macrophage suppressive circuit and resistance to immune checkpoint inhibition, while AHR blockade reversed immune suppression and improved PD‐1 blockade efficacy [33, 186].

Lipid nutrients are also highly immunoregulatory, because lipids support both bioenergetics and suppressive differentiation cues. Under TME nutrient stress, T cells can depend more on mitochondrial FAO, but the solid tumor niche can lock CD8^+^ TILs into persistent ACC activity, promoting lipid synthesis and storage while opposing FAO (including constraints on CPT1A‐dependent mitochondrial fatty acid entry); restricting ACC rewired T cell metabolism and improved energy maintenance and antitumor activity in vivo [102]. Lipid access is also selectively advantageous for suppressive populations: tumor‐infiltrating Tregs used CD36‐mediated adaptation to survive and function in tumors, whereas CD8^+^ T cells experienced CD36‐driven uptake of oxidized lipids that promoted lipid peroxidation and dysfunction [18, 160]. When amino acid constraints overlap with oxidative stress, vulnerability increases: cystine deprivation pushed CD8^+^ TILs toward CD36‐mediated lipid uptake and ferroptosis‐like lipid peroxide accumulation, driving dysfunction that could be mitigated by restoring redox capacity or correcting cystine availability [73]. In myeloid cells, lipid uptake can directly reinforce suppression: FATP2 (SLC27A2) was identified as a driver of PMN‐MDSC suppressive function, linking fatty acid import (including arachidonic acid) to immunosuppressive outputs such as PGE_2_ production [101]. Tumor cells themselves can use lipid metabolism to survive immune attack: cytolytic stress from killer T cells induced tumor CPT1A and FAO in an AMPK‐dependent manner, and CPT1A repression made tumors more susceptible to T cell killing and cellular immunotherapies [99]. Across these nutrient axes, classical nutrient sensors (mTOR, AMPK) and metabolite availability translate into stable functional states partly through epigenetics: acetyl‐CoA supply affects histone acetylation and chromatin accessibility in CD8^+^ T cells, and shifts in nutrient preference in exhausted T cells can create distinct histone acetylation programs at exhaustion‐associated loci [187, 188]. In this way, the “who gets the nutrients” question becomes a continuous tumor–immune crosstalk mechanism that resets immune fate inside the TME.

Overall, amino acid and lipid metabolism jointly function as metabolic checkpoints that dynamically regulate immune cell fate and antitumor activity within the TME.

## 6 Therapeutic Targeting of Amino Acid and Lipid Metabolism

Therapeutic targeting of metabolism is increasingly positioned as an immunotherapy‑sensitizing strategy, because the TME can impose nutrient deprivation, generate immunosuppressive metabolites and lipid mediators, and impair antigen presentation, thereby limiting ICI efficacy [121, 122]. Across both amino acid and lipid pathways, the most consistent translational benefit has been observed in rational combination settings (metabolic intervention plus ICI), whereas setbacks in late‑phase programs highlight pathway redundancy, incomplete pharmacodynamic suppression, and safety limits when biomarker enrichment is lacking [189].

### 6.1 Therapeutic Targeting of Amino Acid Metabolism

#### 6.1.1 Mechanistic Rationale

Amino acids are not only substrates for proliferation but also immune regulatory inputs, because T cells require specific amino acid flux for activation, clonal expansion, and effector differentiation [121, 123, 146]. Within tumors, amino acid restriction can be created by tumor‑intrinsic uptake advantages and by myeloid enzymatic depletion (including arginases and IDO/TDO), which together function as metabolic immune checkpoints [30, 33, 62, 63]. Downstream, kynurenine is an endogenous AHR ligand, enabling suppressive transcriptional programs that further constrain antitumor immunity. Accordingly, therapeutic strategies aim to restore local amino acid availability for lymphocytes, block suppressive catabolic pathways, and preserve immune metabolic flexibility while reducing tumor metabolic dominance [121, 122, 190].

#### 6.1.2 Key Targets and Therapeutic Strategies

##### 6.1.2.1 Glutamine Axis (Transport, GLS, Broad Antagonism/Prodrugs)

Glutamine uptake and utilization support inflammatory T‑cell responses through mTORC1‑linked activation programs, indicating that immune requirements must be considered when targeting this pathway [119]. Meanwhile, tumor‑dominant glutamine programs can support immune evasion and suppressive myeloid phenotypes, motivating combination approaches rather than stand‑alone therapy [119, 121]. GLS1 inhibition (e.g., telaglenastat/CB‑839) has been primarily tested as a combination partner with ICIs, consistent with a sensitization positioning [191]. Broad glutamine antagonism (DON‑derived approaches) is designed to reduce tumor metabolic fitness while allowing immune adaptation under ICI pressure [121]. Tumor‑directed prodrug strategies (e.g., sirpiglenastat/DRP‑104) aim to improve tolerability and tumor selectivity compared with historical broad glutamine antagonists [182, 192].

##### 6.1.2.2 Arginine Axis (Arginase Inhibition; Supportive Arginine Conditioning)

Arginine availability is rate‑limiting for T‑cell metabolic fitness and survival, while arginase‑expressing suppressive myeloid cells can deplete arginine in the TME [30, 146]. Arginase inhibition is intended to restore arginine pools and relieve myeloid‑mediated immune suppression, including in early clinical development alone or combined with PD‑1 blockade [30, 190]. In parallel, arginine conditioning can bias toward persistent, memory‑like T‑cell phenotypes with improved antitumor activity, supporting the logic for “supportive” strategies in adoptive cell therapy contexts [146].

##### 6.1.2.3 Tryptophan/ IDO‐TDO‐AHR Axis (Enzyme Inhibition and Downstream Blockade)

Tryptophan catabolism contributes to immune resistance through tryptophan depletion and kynurenine accumulation, with kynurenine acting as an endogenous AHR ligand that enforces suppressive programs [62, 63]. Clinical experience with IDO1 inhibition‐most notably epacadostat‐demonstrates that mechanistic plausibility does not guarantee benefit, motivating redundancy‑aware strategies [66, 122]. Dual IDO1/TDO2 inhibition has been pursued to address pathway redundancy upstream of kynurenine signaling [193]. AHR inhibition (± PD‑1 blockade) targets downstream signaling more proximally to immune regulation and is therefore positioned as an alternative approach for overcoming kynurenine‑mediated suppression [33, 194].

##### 6.1.2.4 Methionine Axis (Competition, Methylation Programs, Diet Concepts)

Methionine availability shapes epigenetic programming and immune competence, and tumors can outcompete T cells for methionine via transporter‑level mechanisms that impair T‑cell histone methylation and function [131]. Strategies therefore include targeting methionine transport/utilization where selectivity exists and exploring supervised dietary methionine restriction concepts [39, 195].

##### 6.1.2.5 Serine/Glycine Axis (One‑Carbon Metabolism; Immune Expansion Dependency)

Serine is essential for effector T‑cell expansion, meaning that systemic restriction can risk immune impairment if applied indiscriminately [123]. Translationally, strategies are more plausible when immune serine needs are preserved (e.g., targeting tumor one‑carbon dependence or careful scheduling) rather than prolonged global restriction during ICI therapy [158].

##### 6.1.2.6 Asparagine Axis (Activation Signaling; Enzymatic Depletion Strategies)

Asparagine can potentiate CD8 activation and antitumor responses, including via LCK signaling support [74]. Translational evidence also supports that asparagine deprivation can enhance T‑cell antitumor responses via ROS‑mediated metabolic and signaling adaptations, reinforcing context dependence [196].

##### 6.1.2.7 Cystine/cysteine Axis (xCT/SLC7A11; Redox Fitness and Ferroptosis Interface)

Cystine import via SLC7A11 supports glutathione production and redox buffering and represents a vulnerability in defined contexts such as KRAS‑mutant lung adenocarcinoma [197]. Translational strategies focus on tumor‑selective disruption of the xCT/GSH axis and rational combinations that increase tumor oxidative vulnerability while protecting effector lymphocytes from collateral redox injury.

Systemic metabolic toxicities are common constraints (including GI intolerance, hepatic effects, and marrow effects), especially for broad antagonists and restrictive diets [122, 190, 195]. Because immune cells also rely on amino acids, immune suppression is a plausible on‑target risk if dosing is excessive or prolonged, motivating intermittent schedules and pharmacodynamic monitoring [74, 123]. Diet‑based interventions require feasibility safeguards (nutrition monitoring and adherence) and are therefore best positioned as supervised, time‑limited interventions rather than chronic restriction in vulnerable patient populations [195].

### 6.2 Therapeutic Targeting of Lipid Metabolism

#### 6.2.1 Mechanistic Rationale

Lipids regulate antitumor immunity through lipid uptake and storage programs that reprogram DCs and myeloid suppressors, FAO programs that support tumor immune escape, immunosuppressive lipid mediators (COX‑derived PGE2), and lipid peroxidation stress that can disable cytotoxic lymphocytes [18, 91, 101, 110]. Cholesterol handling influences antigen presentation and TCR signaling organization, and lipid post‑translational modifications can stabilize immune checkpoints [93, 108, 118]. Therefore, lipid targeting is most rational as a means to restore antigen presentation, reduce suppressive myeloid activity, and preserve effector CD8 function, typically in combination with immune activation by ICIs [25, 28, 33, 36].

#### 6.2.2 Key Targets and Therapeutic Strategies

##### 6.2.2.1 CD36 (Pathologic Lipid Uptake; Lipid Peroxidation and Dysfunction in CD8 TILs)

Oxidized lipid uptake and CD36‑linked lipid peroxidation can drive CD8 dysfunction in tumors. Strategies include limiting CD36‑dependent lipid import and/or reducing lipid peroxidation stress, typically combined with checkpoint blockade so preserved T‑cell fitness can translate into antitumor activity [18, 91].

##### 6.2.2.2 FATP2 (SLC27A2) in PMN‑MDSC

FATP2 is upregulated in neutrophil‑like suppressor cells and supports suppressive programming via fatty acid uptake and downstream mediator production. Targeting FATP2 aims to reduce suppressor potency and improve ICI responsiveness in myeloid‑dominant tumors [101].

##### 6.2.2.3 FASN (Tumor Lipogenesis; Antigen Presentation/Immune Visibility)

FASN inhibition has been linked to increased MHC‑I stability and improved response to PD‑L1 blockade in preclinical hepatocellular carcinoma, connecting lipogenesis to immune visibility. This positions lipogenesis inhibition as an ICI sensitizer in cancers with strong lipogenic programs and evidence of antigen presentation limitation [95].

##### 6.2.2.4 ACC (T‑Cell Lipid Utilization Constraint Within TME)

ACC can obstruct CD8 lipid utilization and immune killing in the TME, supporting strategies that rewire T‑cell lipid handling toward functional persistence. Because systemic ACC inhibition can have broad metabolic effects, translational strategies require dosing caution and may favor targeted approaches where feasible [102].

##### 6.2.2.5 CPT1A/FAO (Tumor‑Intrinsic Adaptive Resistance)

Tumor CPT1A‑mediated FAO can confer resistance to immune‑mediated cytolytic killing, linking FAO to adaptive immune resistance. Targeting FAO is therefore conceptually attractive but difficult to translate systemically due to immune and metabolic dependencies, motivating combination and tumor‑selective strategies [99].

##### 6.2.2.6 PCSK9 and ACAT1 (Cholesterol Handling, Antigen Presentation, and TCR Signaling)

PCSK9 inhibition potentiates checkpoint therapy in preclinical systems by improving tumor antigen presentation and MHC‑I availability [108]. ACAT1 inhibition can increase membrane cholesterol and enhance TCR clustering, improving CD8 function and antitumor activity in model systems [93]. Safety monitoring must include cardiometabolic domains and immune correlatives given systemic cholesterol biology.

##### 6.2.2.7 COX‐PGE2/EP Receptors (Immune Evasion and cDC1 Dysfunction)

Tumor‑derived PGE2 is a central lipid mediator of immune evasion and can program cDC1 dysfunction that impairs orchestration of antitumor T‑cell responses [110, 111]. Strategies include COX inhibition and EP receptor antagonism (EP4, or EP2/EP4 concepts), often combined with ICIs and ideally timed early to prevent immune exclusion [198, 199, 200].

##### 6.2.2.8 S1P/S1PR Axis (Sphingolipid Signaling)

Sphingosine‑1‑phosphate signaling can metabolically program T cells and limit antitumor activity, supporting investigation of S1P/S1PR modulation as a sensitizer. Because S1P is essential for lymphocyte trafficking and vascular biology, safety constraints are central to translation [115].

##### 6.2.2.9 Palmitoylation (Checkpoint Stability)

PD‑L1 palmitoylation increases PD‑L1 stability, and inhibiting this modification can increase T‑cell immune responses in tumors. Drug development challenges include selectivity and systemic toxicity risk because palmitoylation is widely used [118].

Representative preclinical studies and ongoing clinical trials targeting amino acid and lipid metabolic pathways are summarized in Table 4, organized according to metabolic pathway and mechanism of action. Notably, variability in clinical outcomes highlights the importance of patient stratification, pathway redundancy, and pharmacodynamic optimization in translating metabolic therapies into clinical benefit. In addition, off‐target toxicity, metabolic adaptability of tumors, and context‐dependent immune effects remain major challenges for the clinical application of metabolic therapies.

**TABLE 4:** Clinical trials targeting Amino acid and lipid metabolism.

Pathway	Agent	Target/pathway	Phase	Indication	Primary endpoints	Status/results (if available)	Study IDs
Glutamine	Telaglenastat (CB‑839) + nivolumab	GLS1 + PD‑1	I/II	Melanoma/RCC/NSCLC cohorts	Safety; activity by cohort	Published: tolerable; mixed activity	NCT02771626
Glutamine (prodrug)	Sirpiglenastat (DRP‑104) ± atezolizumab	Broad glutamine antagonism ± PD‑L1	I/II	Advanced solid tumors	Safety/PK/PD; preliminary activity	Registry ongoing; peer‑reviewed outcomes pending	NCT04471415
Arginine	INCB001158 ± pembrolizumab	Arginase inhibition ± PD‑1	I	Advanced solid tumors	Safety (primary); PK/PD; activity	Published: tolerable; PD activity; limited objective responses typical for phase I	NCT02903914
Tryptophan	Epacadostat + pembrolizumab	IDO1 + PD‑1	I/II	Advanced solid tumors	Safety/MTD; PK; early activity	Published early‐phase results	ECHO‐202/KEYNOTE‐037
Tryptophan	Epacadostat + pembrolizumab vs placebo + pembrolizumab	IDO1 + PD‑1	III	Advanced melanoma	PFS/OS	Published negative phase III	NCT02752074
Tryptophan	Navoximod + atezolizumab	IDO1 + PD‑L1	I	Advanced solid tumors	Safety/PK/PD	Published: limited responses	N/A
Tryptophan	M4112	Dual IDO1/TDO2	I	Advanced solid tumors	Safety/PK/PD; preliminary efficacy	Published first‐in‐human	N/A
Tryptophan/AHR	IK‑175 ± nivolumab	AHR inhibitor ± PD‑1	I/lb	Solid tumors; UC expansion	Safety; target engagement; activity	Published: target engagement; preliminary activity	NCT04200963
COX‐PGE2/EP4	E7046	EP4 antagonist	I	Advanced cancers	Safety/PK/PD	Published phase I	NCT02540291
COX‐PGE2/EP4	AN0025 + total neoadjuvant therapy	EP4 antagonist	Ib	Locally advanced rectal cancer	Safety; response signal	Published: response signal	N/A
COX inhibitors + ICI	PD‑1 blockade + COX inhibitors (PCOX)	COX pathway + PD‑1	II	dMMR metastatic CRC	ORR	Published: high ORR and biomarker analyses	NCT03638297
EP4 antagonist + ICI	ONO‑4578 + nivolumab	EP4 antagonist + PD‑1	I	Gastric/GEJ cancer	Safety; preliminary efficacy; biomarkers	Published early activity signal	NCT03155061
Cholesterol/PCSK9	PCSK9 inhibitor + ICI	PCSK9 + ICI	II	NSCLC (examples)	Safety/ORR/PFS + immune correlatives	Registry programs exist; results pending	NCT05144529/NCT05553834

Abbreviations: AHR, aryl hydrocarbon receptor; ARG1, arginase 1; CB‐1158, telaglenastat/arginase inhibitor (small molecule class inhibitor used in clinical studies); CD8^+^ T, CD8‐positive T lymphocyte; CRC, colorectal cancer; DC, dendritic cell; dMMR, mismatch repair deficient; EPACADOSTAT, IDO1 inhibitor; FAO, fatty acid oxidation; FATP2, fatty acid transport protein 2; GEJ, gastroesophageal junction; GLS1, glutaminase 1; HCC, hepatocellular carcinoma; ICI, immune checkpoint inhibitor; IDO1, indoleamine 2,3‐dioxygenase 1; Kyn, kynurenine; MDSC, myeloid‐derived suppressor cell; MHC‐I, major histocompatibility complex class I; MTD, maximum tolerated dose; N/A, not available; NK, natural killer cell; NSCLC, non‐small cell lung cancer; ORR, objective response rate; OS, overall survival; PD, pharmacodynamics; PD‐L1, programmed death‐ligand 1; PFS, progression‐free survival; PK, pharmacokinetics; RCC, renal cell carcinoma; SAM, S‐adenosylmethionine; TAM, tumor‐associated macrophage; TCA, tricarboxylic acid cycle; TDO, tryptophan 2,3‐dioxygenase; TME, tumor microenvironment; Trp, tryptophan; Treg, regulatory T cell; UC, urothelial carcinoma.

*Data sources: ClinicalTrials.gov*.

## 7 Challenges, Limitations, and Future Perspectives

Although amino acid and lipid metabolism have become important fields in cancer research, several challenges still limit their clinical translation. First, most current studies focus on single metabolic pathways, such as glutamine metabolism, arginine depletion, tryptophan–kynurenine–AHR signaling, FAO, cholesterol metabolism, or ferroptosis. However, tumor metabolism is not controlled by one isolated pathway. Amino acid and lipid metabolism are closely connected through carbon flux, redox balance, mitochondrial activity, epigenetic regulation, and shared regulators such as mTOR, MYC, AMPK, and HIF‐1α [201, 202]. Therefore, inhibition of one pathway may be compensated by another metabolic program. This metabolic plasticity partly explains why some agents show strong preclinical activity but limited clinical benefit [203].

Second, metabolic pathways are highly context‐dependent. The same metabolic process may have opposite effects in tumor cells and immune cells. For example, glutamine metabolism can support tumor growth, but it is also required for T cell activation and DC function [124]. Lipid uptake and FAO may promote suppressive myeloid cells or tumor resistance, but they can also be important for some effector immune cell states [101]. Ferroptosis is another typical example, because lipid peroxidation may kill tumor cells but may also damage CD8^+^ T cells or other immune cells when antioxidant protection is insufficient [91]. These findings indicate that metabolic therapy should not simply aim to block one nutrient or one enzyme, but should consider the dominant cellular compartment, treatment timing, tumor type, and immune status.

Third, the lack of reliable biomarkers remains a major limitation. Many clinical trials still use broad patient populations without clear metabolic stratification. Future studies should include metabolomics, lipidomics, single‐cell sequencing, spatial transcriptomics, imaging mass cytometry, and immune profiling to identify which patients are most likely to benefit from metabolic intervention. Biomarkers may include transporter expression, enzyme activity, metabolite accumulation, immune cell infiltration, antigen‐presentation status, lipid peroxidation level, and signatures of nutrient competition. Such integrated analysis may also help distinguish whether a metabolic pathway is mainly tumor‐promoting, immune‐suppressive, or immune‐supportive in a specific tumor context.

Fourth, toxicity and physiological metabolic dependence need careful consideration. Amino acids and lipids are essential for normal tissues, and systemic inhibition may affect immune cells, liver metabolism, muscle function, intestinal homeostasis, and general nutritional status. Dietary restriction, microbiome‐based regulation and metabolic inhibitors may provide useful strategies, but they need precise control and clinical validation. In this regard, tumor‐selective prodrugs, local delivery systems, intermittent treatment schedules, and rational combination regimens may help improve the therapeutic window.

Instead of metabolic monotherapy, emerging trends suggest that the most promising direction is rational combination therapy. Metabolic interventions may enhance immune checkpoint blockade, adoptive cell therapy, radiotherapy, or targeted therapy by reducing immunosuppressive metabolites, improving antigen presentation, restoring T cell function, and weakening suppressive myeloid programs [108, 121]. In the future, dual targeting of amino acid and lipid metabolism may be particularly valuable, because these two metabolic axes jointly regulate nutrient competition, immune cell fitness, and tumor adaptation under therapeutic pressure. More mechanism‐based preclinical models and biomarker‐driven clinical trials are required to determine how to select patients, combine drugs, avoid immune damage, and translate metabolic vulnerabilities into durable antitumor responses.

## 8 Conclusion

Amino acid and lipid metabolism are central components of cancer metabolic reprogramming and have broad effects on tumor progression, immune escape, and therapeutic resistance. Amino acids provide substrates for biosynthesis, redox balance, epigenetic regulation, and immune cell activation, whereas lipid metabolism supports membrane formation, energy production, signaling, immune checkpoint regulation, and ferroptosis‐related cell fate. These pathways do not work separately, but form an interconnected metabolic network within the TME.

Importantly, amino acid and lipid metabolism also regulate antitumor immunity by shaping nutrient competition, antigen presentation, myeloid cell polarization, T cell exhaustion, and response to immune checkpoint blockade. Therefore, metabolic intervention should be considered not only as a tumor‐targeting strategy, but also as a way to remodel tumor–immune interactions. A better understanding of tumor‐ and immune‐cell‐specific metabolic dependencies will help identify actionable vulnerabilities and support rational combination therapies. Overall, integrated targeting of amino acid and lipid metabolism may provide new opportunities to improve precision cancer immunotherapy.

## Author Contributions

Z.W. wrote the draft and made the figures. X.F. revised the manuscript and wrote the lipid metabolism part of the manuscript. J.B. helped with the revised figures and tables. Y.L. and K.H. initiated the idea and edited the manuscript. All authors agreed on the submission of the manuscript.

## Funding

This work is funded by National Natural Science Foundation (NSF) of China (No. 82103209), Natural Science Foundation of Shandong Province (ZR2024QH044), the “20 New Higher Education Initiatives” Research Leader Studio Project of Jinan Science & Technology Bureau (No. 202228122), the Shandong Provincial Medical Association (No. YXH2022DZX02004), and The Science & Technology Development Fund of Tianjin Education Commission for Higher Education (2022KJ230, NO.21JCYBJC01450).

## Ethics Statement

The authors have nothing to report.

## Conflicts of Interest

The authors declare no conflicts of interest.

## Data Availability

The authors have nothing to report.
